# Inborn Errors of Immunity Presenting with Early-Onset Severe Atopy

**DOI:** 10.3390/medicina61010062

**Published:** 2025-01-02

**Authors:** Nipat Chuleerarux, Nadia Makkoukdji, Travis Satnarine, Jessica Elise Kuhn, Tanawin Nopsopon, Peerada Valyasevi, Fernanda Bellodi Schmidt, Gary Kleiner, Melissa Gans

**Affiliations:** 1 Department of Internal Medicine, Jackson Memorial Hospital, University of Miami Miller School of Medicine, Miami, FL 33136, USA; 2 Department of Pediatrics, Jackson Memorial Holtz Children’s Hospital, University of Miami Miller School of Medicine, Miami, FL 33136, USA; 3 Division of Allergy and Clinical Immunology, Brigham and Women’s Hospital, Boston, MA 02115, USA; 4 Faculty of Medicine, Chulalongkorn University, Bangkok 10330, Thailand; 5 Dr. Phillip Frost Department of Dermatology and Cutaneous Surgery, University of Miami Miller School of Medicine, Miami, FL 33136, USA; 6 Division of Allergy/Immunology, Department of Pediatrics, Jackson Memorial Holtz Children’s Hospital, University of Miami Miller School of Medicine, Miami, FL 33136, USA

**Keywords:** inborn errors of immunity, primary immune deficiencies, atopy, atopic dermatitis

## Abstract

Inborn errors of immunity (IEIs), also known as primary immunodeficiencies, are a group of genetic disorders affecting the development and function of the immune system. While IEIs traditionally present with recurrent infections, an increasing number of cases manifest with early-onset severe atopy, including atopic dermatitis, food allergies, asthma, and allergic rhinitis—features that are often overlooked. This can lead to delayed diagnosis and treatment, which is crucial for IEI patients due to the risk of severe infections. We conducted a literature search and reviewed all IEIs that can present with early-onset severe atopy. The hallmark features of these disorders often include early-onset, persistent, and severe atopic dermatitis, food allergies, and recurrent episodes of asthma, which may be refractory to treatments. Additionally, we discuss the importance of recognizing such severe atopy as a potential indicator of an underlying immune deficiency, particularly when accompanied by unusual infections, growth failure, or autoimmunity. This review aims to raise awareness of this association and emphasize the need for early diagnosis and genetic testing in patients with atypical or treatment-resistant allergic diseases, allowing for more timely diagnosis of underlying immunodeficiencies and appropriate treatments.

## 1. Introduction

Inborn errors of immunity (IEIs), also known as primary immunodeficiencies, are a diverse group of genetic disorders that result in the dysfunction of the immune system [1]. These disorders can lead to a wide range of clinical manifestations, including increased susceptibility to infections, autoimmunity, and malignancies [1]. IEIs can also present with early-onset atopy, which includes allergic conditions such as asthma, atopic dermatitis, and allergic rhinitis [1].

The association between IEIs and severe atopic manifestations in early childhood suggests a complex interplay between genetic variants and immune system dysregulation [2]. For example, defects in immune regulatory pathways can lead to an exaggerated Th2 immune response [3]. The recognition of IEIs presenting with atopy is challenging, as some IEIs may present with symptoms resembling allergic diseases [4]. This can lead to delayed diagnosis or misdiagnosis, delaying proper treatments, which can significantly impact patient outcomes [5]. Additionally, not identifying the allergic components of certain clinical manifestations in IEI patients can exacerbate their condition and prevent them from receiving appropriate allergy treatments, such as biologics [4].

This review aims to explore the intersection of IEIs and severe early-onset atopy, focusing on genetic defects, immunological mechanisms, clinical implications, and management. By reviewing recent findings, we seek to enhance the understanding of these complex conditions and highlight the different clinical manifestations of IEIs and potential strategies for management and intervention.

### 1.1. Inborn Errors of Immunity and Early-Onset Severe Atopy

Inborn errors of immunity (IEIs) are a diverse group of genetic disorders characterized by defects in the immune system that lead to increased vulnerability to infections, autoimmunity, inflammatory diseases, malignancy, and allergy [1]. Over 450 distinct IEIs have been identified, affecting various components of the immune response, including antibodies, T cells, B cells, phagocytes, and the complement system [1]. These conditions range in severity from mild to life threatening, often manifesting early in life [1]. While recurrent infections are the hallmark of IEIs, there is a growing recognition of their association with allergic diseases, or atopy [4].

Atopy refers to the genetic predisposition to develop allergic reactions and is characterized by an exaggerated immune response to common environmental allergens [4]. Early-onset atopy lacks a clear definition, but some studies describe it as the appearance of atopic symptoms within the first year of life [6,7]. Th2 cells are essential components of the type II immune response, which is involved in typical atopic diseases [8]. This response is crucial for protection against helminth infections, supporting tissue repair, neutralizing toxins, and regulating various inflammatory processes [8]. Th2 cells secrete cytokines, such as IL-4, IL-5, IL-9, and IL-13, which recruit and activate mast cells, eosinophils, and basophils and induce B cell class switching to produce IgE antibodies [9,10,11]. An inability to properly control Th2 cells or their downstream targets can lead to clinical symptoms associated with allergic and atopic diseases, such as IgE-mediated hypersensitivity, mast cell activation, allergic inflammation of the skin and mucosa, and interference with other inflammatory responses [12]. The most common atopic disorders include asthma, allergic rhinitis, atopic dermatitis, and food allergy, each of which can significantly impact the quality of life due to chronic symptoms such as pruritus, sneezing, bronchoconstriction, and skin inflammation [13,14].

The connection between IEIs and atopy suggests that immune dysregulation can lead not only to impaired pathogen defense but also to hypersensitivity to environmental antigens [15]. This overlap is particularly evident in disorders, such as *DOCK8* deficiency and Wiskott–Aldrich syndrome, where patients frequently present with both immunodeficiency and severe atopic manifestations [15,16]. Understanding the dual nature of IEIs in promoting susceptibility to infections and atopic disorders is crucial for accurate diagnosis and effective management, enabling clinicians to tailor treatment strategies that address both aspects of these complex conditions. In this review, we categorize IEIs presenting with early-onset severe atopy by genes based on their functions. Clinical manifestations, investigations, and management are summarized in Table 1.

### 1.2. T Cell Signaling

#### 1.2.1. *CARD11* GOF/LOF/DN

**Figure 1:**
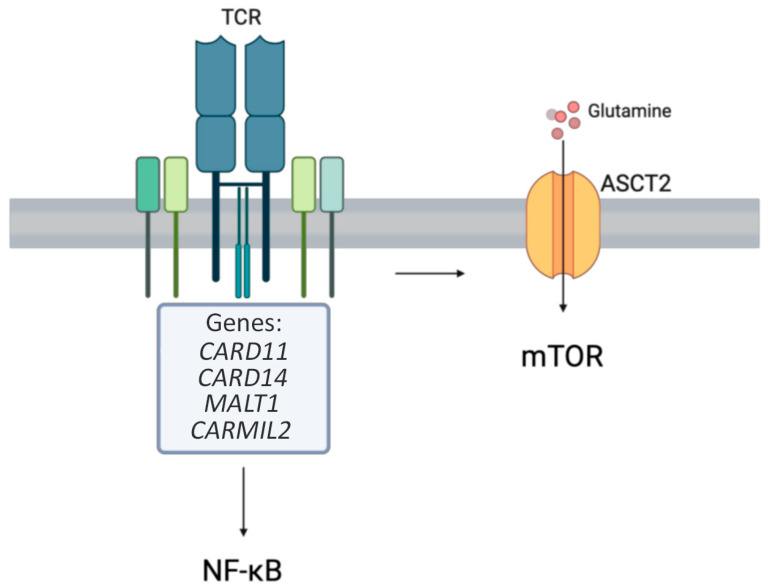
The CBM complex controls T cell signaling by the mammalian target of rapamycin (mTOR), nuclear factor kappa B (NF-κB), and c-Jun N-terminal kinase activation (JNK). The T cell receptor leads to the transportation of glutamine, initiating the mTOR pathway.

*CARD11*, a caspase recruitment domain-containing protein, is crucial for immune cell signaling [17]. It plays a pivotal role in the activation of nuclear factor kappa B (NF-κB), c-Jun N-terminal kinase (JNK), and mammalian target of rapamycin (mTOR) pathways (Figure 1) [17]. Pathogenic variants in *CARD11* can result in gain-of-function (GOF), loss-of-function (LOF), or dominant-negative (DN) alterations, affecting immune responses differently [17].

GOF variants in *CARD11* are associated with an autosomal dominant condition called BENTA (B cell expansion with NF-κB and T cell anergy), which leads to lymphoproliferation, immune dysregulation, and atopy in some cases [18]. Atopy is less common in BENTA than other *CARD11* diseases and is not necessarily early onset. However, in one patient described with BENTA, asthma was mentioned as a manifestation, but the age at which it occurred was not detailed [18].

LOF variants in *CARD11* are associated with a condition called CADINS (*CARD11*-associated atopy with dominant interference of NF-kB signaling), which is linked to severe atopic diseases, such as atopic dermatitis, and other immune deficiencies [17]. The mean age of disease onset for patients with LOF variants (CADINS) was reported as 5.9 months of age [18]. Atopy develops early in individuals with dominantly inherited hypomorphic LOF variants in *CARD11*, often presenting as severe atopic dermatitis in childhood, sometimes associated with other allergic manifestations such as asthma, elevated IgE levels, and eosinophilia [18]. These *CARD11* variants lead to impaired signaling pathways, particularly in NF-κB and mTORC1, which contribute to the immune dysfunction seen in these patients. The variants cause a Th2-skewed immune response, predisposing individuals to severe allergic disease [19].

DN variants in *CARD11*, a scaffold protein crucial for linking antigen receptor signaling to downstream immune pathways, result in impaired NF-κB and mTORC1 activation [20]. These variants are associated with a broad spectrum of immune phenotypes, including severe atopic diseases, viral skin infections, hypogammaglobulinemia, neutropenia, and even lymphoma, with atopy typically presenting in childhood, especially as atopic dermatitis [20]. DN *CARD11* variants are associated with a wide spectrum of immune system presentations, extending beyond atopy and high IgE levels [20]. These variants can result in a combined immunodeficiency manifesting as recurrent viral skin infections, hypogammaglobulinemia, neutropenia, and in some cases, lymphoma [20]. The severity and combination of symptoms vary, but most patients exhibit atopy, while others may present with more severe immunologic disorders without the typical atopic features [21].

Differentiation from intrinsic atopy involves comprehensive genetic testing and immunophenotyping to identify specific immune cell defects [19,22]. Diagnosis involves genetic testing for *CARD11* variants, immunophenotyping to assess T and B cell subsets, and the measurement of serum immunoglobulin levels. Functional assays of immune cells may be conducted to evaluate the activation of NF-κB and other signaling pathways [23]. Management includes replacement immunoglobulin, aggressive treatment of infections, and treatment of dermatitis and other atopic conditions [22]. Experimental approaches, like glutamine supplementation, have shown the potential to correct some of the defective signaling pathways [19,22].

#### 1.2.2. *CARD14* GOF/LOF

*CARD14* GOF variants are linked to inflammatory skin disorders, particularly a unique combination of features resembling psoriasis and pityriasis rubra pillars, which is known as *CARD14*-associated papulosquamous eruption (CAPE) [24]. *CARD14*, similar to *CARD11*, activates NF-κB signaling but in keratinocytes, contributing to skin inflammation and hyperplasia (Figure 1) [23]. *CARD14* GOF variants, particularly the PSORS2 single-nucleotide polymorphism (SNP), are major genetic risk factors for psoriasis, leading to enhanced NF-kB activation, the upregulation of psoriasis-associated genes in keratinocytes, and triggering acute inflammatory responses and recruitment of inflammatory infiltrate, making *CARD14* a key regulator of skin inflammation [25]. Biopsies of CARD14-associated papulosquamous eruptions show alternating checkerboard parakeratosis and orthokeratosis, acanthosis without acantholysis, dilated dermal papillae vessels, and occasional follicular plugging [26]. Though psoriasis and atopic dermatitis are both T cell-mediated inflammatory skin conditions, psoriasis is not classically viewed as an atopic disease [27]. However, *CARD14* LOF variants have been reported to be associated with severe atopic dermatitis [28]. The onset of atopic dermatitis was reported to occur as early as 3 months of age [28]. Management focuses on controlling skin inflammation, and success has been achieved through the use of ustekinumab [29,30].

#### 1.2.3. *MALT1* LOF

*MALT1* encodes a caspase-like protease involved in BCL10-induced NF-kB activation, which is part of the CARMA1-BCL10-MALT1 signalosome for NF-kB signaling and lymphocyte activation (Figure 1) [31]. Autosomal recessive variants in this gene cause MALT1 deficiency with recurrent rearrangements in mucosa-associated lymphoid tissue lymphomas involving specific chromosomal translocations [32]. *MALT1* deficiency has been associated with the development of atopic disease, potentially due to a skewing toward Th2 immune responses as a result of impaired MALT1 paracaspase activity [33]. The onset of atopy in patients with *MALT1* variants may be as early as 2 weeks of age in early infancy [34]. Similar patterns have been observed in cases of impaired *MALT1* function, leading to features such as dermatitis and recurrent infections (bacterial, viral, and fungal in addition to periodontal disease, enteropathy, and failure to thrive) [33]. The impaired Th17 pathway in *MALT1* LOF disease likely causes these severe and recurrent infections [33]. Investigations usually show elevated IgE levels and eosinophilia [33]. The treatment for *MALT1* LOF disease is hematopoietic stem cell transplantation (HSCT) [35].

#### 1.2.4. *CARMIL2* LOF

*CARMIL2* deficiency is an autosomal recessive disorder characterized by defective CD28-mediated T cell receptor (TCR) co-signaling and impaired cytoskeletal dynamics, leading to a lack of regulatory T cells and issues with T cell activation, differentiation, and function [36]. Four patients with EBV+ disseminated smooth muscle tumors, and two homozygous LOF variants in the *CARMIL2* gene exhibited these deficiencies without organ-specific autoimmunity [36]. Seven patients from three unrelated consanguineous families with combined immunodeficiency have been described with dermatitis, esophagitis, and recurrent skin infections [37]. These patients demonstrated reduced Treg counts, skewed naïve CD4+ T cells, and diminished CD3/CD28 signaling [37]. The onset of dermatitis in some patients with *CARMIL2* LOF disease can be early and severe, as seen in a 4-year-old boy who exhibited eczematous dermatitis at 2 weeks of age [37]. Patients may have also elevated IgE levels and an inadequate response to vaccinations [37]. Management of *CARMIL2* deficiency includes immunoglobulin replacement therapy, careful monitoring for EBV+ smooth muscle tumors, and considering HSCT as a potential cure [38].

### 1.3. JAK/STAT Pathway 

#### 1.3.1. *STAT1* GOF

**Figure 2:**
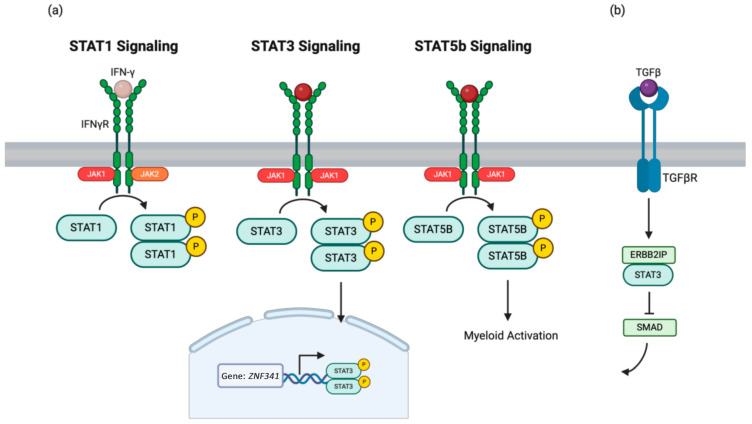
(**a**) The JAK/STAT pathways contain various factors, including *STAT5,* which leads to myeloid activation, or *STAT3*, which upregulates transcription of the gene *ZNF341*. (**b**) TGF-β signaling activates ERBIN/STAT3 complexes, inhibiting SMAD transport into the cell nucleus.

GOF variants in *STAT1* (*STAT1* GOF) lead to hyperactive *STAT1* signaling (Figure 2a), resulting in chronic mucocutaneous candidiasis and increased susceptibility to viral infections [39]. These variants enhance responses to IFN-γ and other cytokines, leading to impaired Th17 cell differentiation and increased apoptosis of immune cells [40]. Patients with heterozygous *STAT1* GOF variants often present with atopy, dermatitis, and recurrent mucocutaneous fungal infections, predominantly affecting the skin, nails, and mucosae [39]. The median age of onset is one year [39]. Diagnostic investigations include genetic testing for *STAT1* variants, immunological profiling revealing low IL-17A-producing T cells, and comprehensive microbiological assessments to identify fungal, bacterial, and viral infections [39]. Management involves considering long-term antifungal therapy and antibacterial prophylaxis, and in severe cases, JAK inhibitors or HSCT for refractory infections with autoimmune complications [39].

#### 1.3.2. *STAT3* DN

DN variants in *STAT3* (*STAT3* DN) result in a compromised immune response [41]. *STAT3* is a critical transcription factor for various cellular processes, including cytokine signaling, cell growth, and apoptosis (Figure 2a) [42]. Variants lead to impaired signaling pathways, affecting Th17 cell differentiation and IL-6-mediated responses, which are essential for fighting bacterial and fungal infections [42]. Atopy may present early in life in patients with *STAT3* DN disease, which is also known as STAT3 hyper-IgE syndrome (*STAT3*-HIES) [41]. Manifestations can include a newborn papulopustular rash resembling neonatal acne, occurring in approximately 50% of patients within the first two weeks of life [41]. This rash progresses into atopic dermatitis, which is a key atopic feature of the syndrome. However, the specific age of onset for atopy can vary, though it typically begins in infancy [41]. Diagnosis of *STAT3* DN disease involves genetic testing for *STAT3* variants and laboratory analysis, including elevated serum IgE levels and the absence of IL-17-producing Th17 lymphocytes [41]. Management focuses on aggressive antimicrobial prophylaxis and treatment to prevent infections and end-organ complications, with emerging but uncertain potential for HSCT [41].

#### 1.3.3. *STAT5b* LOF

*STAT5b* enables DNA-binding transcription activator and RNA polymerase II-specific activities, is involved in various cellular responses and regulatory pathways, affects organ development and lymphocyte activation, is located in the cytoplasm and nucleus, and is expressed in multiple body structures, and its human orthologs are implicated in growth hormone insensitivity syndromes with immune dysregulation (Figure 2a) [43]. Patients with *STAT5b* deficiency may present with chronic atopic dermatitis and other atopic conditions, alongside immune dysregulation and growth hormone insensitivity [44,45]. The onset of symptoms is early in life [46,47]. Diagnostic evaluations include sequencing to identify *STAT5b* variants. Patients were commonly found to have low insulin-like growth factor 1, hypergammaglobulinemia, and low T cell counts [48]. Management strategies focus on addressing immune dysregulation with immunosuppressive therapies including steroids, optimizing skin care for dermatitis, and potentially hormone therapy for growth hormone insensitivity [47,49].

#### 1.3.4. *STAT5b* GOF

*STAT5b* autosomal dominant GOF variants lead to increased STAT5 signaling, which results in clonal dominance and maintenance by providing a proliferation advantage to the mutated cells [43]. This heightened signaling causes dysregulated immune responses, including elevated Th2 cytokine production and decreased Th1 cytokine production, contributing to higher lymphocyte and large granular lymphocyte counts in CD4+ T cell large granular lymphocytic leukemia patients compared to wild-type patients [50,51]. Patients can present with severe, treatment-resistant atopic dermatitis, chronic spontaneous urticaria, and hypereosinophilia, with some experiencing alopecia, angioedema, and recurrent gastrointestinal issues [50,52]. Symptoms can begin as early as a few months of age [52,53]. Diagnostic workup includes genetic sequencing to identify *STAT5B* variants, immunophenotyping of blood cells, and cytokine response assays [50]. Management focuses on controlling inflammation and immune dysregulation with JAK inhibitors, addressing skin conditions, and in severe cases, considering HSCT [50].

#### 1.3.5. *JAK1* GOF

The GOF variant in the *JAK1* gene (*JAK1* GOF) causes constitutive activation of the JAK1 protein kinase, disrupting immune regulation and enhancing myelopoiesis, which leads to increased eosinophils and other myeloid-derived cells [54]. These pathogenic variants skew T-helper cell differentiation towards a Th2 phenotype, increasing IL-4, IL-13, and IFN production, exacerbating allergic responses, and resulting in severe eosinophilia and multiple allergic conditions, like severe atopic dermatitis, asthma, and food allergies, in addition to failure to thrive, autoimmune thyroiditis, elevated eosinophil counts, gastrointestinal disease, and hypothyroidism [15,54,55]. One patient with *JAK1* GOF disease developed food allergies, specifically a fish allergy, by the age of 2 [54]. Genetic sequencing identifies the *JAK1* GOF variant, and further analysis using induced-pluripotent stem cells (iPSCs), zebrafish models, and RNA-Seq can reveal dysregulated genes involved in IL-4, IL-13, and IFN signaling [54]. Long-term treatment with a JAK inhibitor can significantly improve allergic symptoms, eosinophilia, and growth in affected children [54].

#### 1.3.6. *ZNF341* LOF

The *ZNF341* gene encodes a transcription factor that regulates the *STAT1* and *STAT3* genes and is crucial for immune system function, particularly T cell and B cell maturation, and it is also involved in bone and tissue development (Figure 2a) [56]. Patients with *ZNF341* deficiency often present with atopic dermatitis, recurrent skin infections, and chronic mucocutaneous candidiasis [57]. Atopic dermatitis can occur as early as 2 years of age [57]. Diagnostic investigations include genetic testing to identify biallelic autosomal recessive LOF variants in the *ZNF341* gene. Patients typically exhibit similar immunological phenotypes to those with *STAT3* deficiency, including elevated IgE levels and eosinophilia [57]. Management strategies are largely supportive and can include controlling atopic dermatitis, treating infections promptly with antibiotics or antifungals, and monitoring and managing associated immunodeficiencies [57].

### 1.4. NF-κB

#### 1.4.1. *RelB* LOF

**Figure 3:**
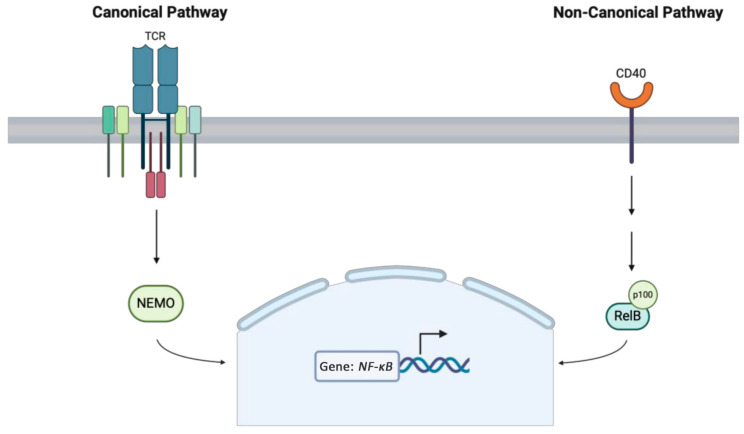
NF-κB signaling pathway, involving either NEMO in the canonical pathway or *RelB* in the non-canonical pathway, is crucial for cell proliferation and immunity.

The *RelB* gene encodes RelB, a key NF-κB transcription factor (Figure 3) [58]. Several deleterious variants in this gene resulting in the downregulation of *RelB* have been associated with an autosomal recessive combined immunodeficiency (CID) [58]. *RelB* deficiency has been identified in a small cohort of patients presenting with T and B cell dysfunction or depletion, recurrent bacterial infections, failure to thrive, and autoinflammation with onset in infancy [58]. *RelB* deficiency has also been linked with the development of T cell-dependent skin disease resembling atopic dermatitis and parabronchial inflammation, as seen in asthma [59]. The mainstay treatment involves immunoglobulin replacement and antibiotic prophylaxis; however, several cases of successful cures with HSCT have been reported [60].

#### 1.4.2. *NF-κB1* LOF

*NF-κB1* encodes a protein that is a crucial component of the DNA binding subunit of the NF-κB protein complex, which is vital for inflammation, immunity, and cell proliferation (Figure 3) [61]. Autosomal dominant LOF variants in *NF-κB1* are widely reported as the most common monogenic cause of common variable immunodeficiency [61]. The clinical features and age of onset in affected individuals vary widely but typically include hypogammaglobulinemia, recurrent respiratory infections, EBV proliferation, and autoimmunity [61]. Skin manifestations are common, including recurrent skin infections, autoimmune skin conditions, and severe atopic dermatitis [61]. Treatment for *NF-κB1* deficiency depends on the specific phenotype [61]. Those with primary antibody deficiency are usually treated with immunoglobulin replacement [61]. Primary autoimmune presentations are managed with systemic steroids or other immunosuppressants. The role of HSCT in managing this disease remains controversial [61].

#### 1.4.3. *IKBKG* LOF

*IKBKG* encodes NEMO, a subunit of the IKK complex which is a key regulatory complex in the activation of NF-κB (Figure 3) [62]. Anhidrotic ectodermal dysplasia with immunodeficiency (EDA-ID) is a rare IEI caused by both X-linked recessive and autosomal dominant LOF variants in *IKBKG* [62]. The phenotype is variable, but EDA-ID is characterized by hypodontia, hypohidrosis, hypotrichosis, atopic dermatitis-like skin eruption, and abnormal facial features soon after birth [62]. Immunologic findings include susceptibility to opportunistic infections, including viral, bacterial (particularly mycobacterium), and fungal infections, in addition to hypogammaglobulinemia and impaired natural killer (NK) cell activity [62]. EDA-ID is potentially curable with HSCT though with variable outcomes [63].

### 1.5. Cytoskeletal Pathway

#### 1.5.1. *WAS* LOF

**Figure 4:**
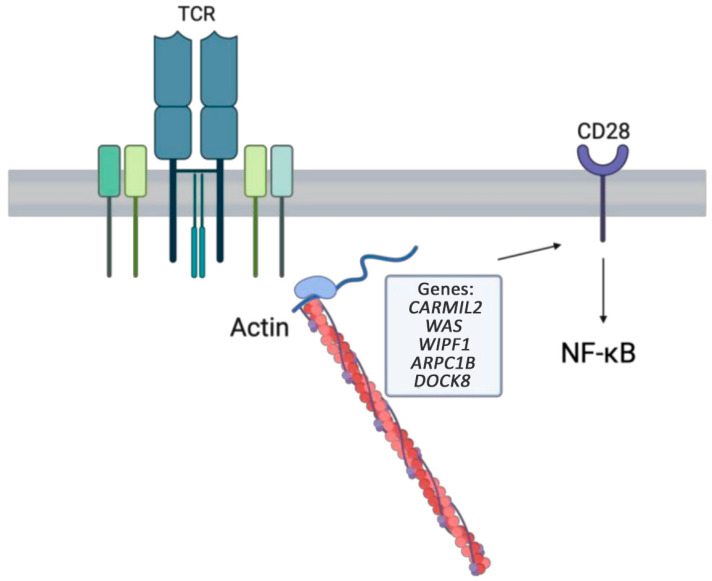
The cytoskeletal pathway activated via a T cell receptor contributes to actin assembly, which is necessary for regulatory T cell functioning, intracellular interactions, and NF-κB signaling.

One key regulator of the actin cytoskeleton is the Wiskott–Aldrich syndrome protein (WASP) [64]. The WASP is encoded by the *WAS* gene localized on the X chromosome and is exclusively expressed in hematopoietic cells [64]. The WAS protein is present in non-erythroid hematopoietic cells, where it acts as a connector between signaling pathways and the movement of actin filaments within the cytoskeleton (Figure 4) [65]. This component of the cellular structure is essential for intracellular interactions, cell–substrate interactions, and signaling due to its role in determining cell shape and facilitating cell movement [65]. The classic presentation of WAS includes thrombocytopenia, severe atopic dermatitis, recurrent infections (particularly EBV, HSV, and periodontal disease), defective T cell proliferation and chemotaxis, and impaired natural killer cell function, in addition to an increased incidence of autoimmunity and lymphoma [66]. The dermatitis seen in WAS is similar to intrinsic atopic dermatitis, except for the presence of petechiae, which can result from minimal trauma, such as scratching the affected areas, as well as ecchymosis and a tendency for secondary infections [67]. Atopic dermatitis usually presents within the first year of life [67]. Milder phenotypes also exist, including X-linked thrombocytopenia and X-linked neutropenia, which are associated with a more normal WASP expression [68,69]. The treatment of choice in patients with severely affected WAS is HSCT due to the high risk of death from thrombocytopenia and treatment-resistant lymphoma [70].

#### 1.5.2. *WIPF1* LOF

The *WIPF1* gene encodes WIP, which is fundamental to WASP stabilization, and the DOCK8-WIP-WASP complex that links TCRs to the actin cytoskeleton (Figure 4) [71]. A novel stop codon variant in the *WIPF1* gene has been identified to cause Wiskott–Aldrich syndrome protein-interacting protein (WIP) deficiency [72]. Clinically, WIP deficiency presents similarly to WAS with recurrent infections, severe dermatitis, thrombocytopenia, and T cell and NK cell dysfunction [72]. Atopic dermatitis can present as early as 11 days of age [72]. Distinct from classical *WAS*, in patients with aberrant *WIPF1*, cells have undetectable *WAS* protein but a normal *WAS* sequence and mRNA [72]. HSCT is the only definitive treatment modality; however, patients may be bridged with antibiotic, antifungal, and antiviral prophylaxis as well as serial transfusions [73].

#### 1.5.3. *ARPC1B* LOF

The *ARPC1B* gene encodes a key subunit of the human actin-related protein 2/3 complex, which is implicated in the control of actin polymerization primarily in hematopoietic cells (Figure 4) [74]. A newly described IEI likened to *WAS* has been attributed to novel *ARPC1B* LOF variants [75]. ARBC1B deficiency is an autosomal recessive CID characterized by impaired T cell migration and proliferation, anaphylactic food allergies, atopic dermatitis, asthma, and thrombocytopenia [75,76]. To date, few patients have been diagnosed with *ARPC1B* deficiency [75,76]. One patient presented with atopic dermatitis, food allergies, and asthma as early as 6 months of age [75]. Elevated IgE levels were found in these patients [75]. Current evidence supports HSCT as the only curative treatment modality in patients with this diagnosis [77].

#### 1.5.4. *DOCK8* LOF

*DOCK8* plays a crucial role in organizing the cytoskeleton (Figure 4). Deficiency in this protein disrupts dendritic cell movement, reduces T cell survival, and impairs NK cell functioning [71]. Variants in *DOCK8* lead to an autosomal dominant CID characterized by early-onset elevated IgE, atopic dermatitis, asthma, severe viral skin infections, bacterial respiratory infections, severe food or environmental allergies, including anaphylaxis, and a predisposition to malignancy [78]. Early HSCT is gaining prominence as a definitive treatment for patients with *DOCK8* variants [79]. Dupilumab has been used to bridge patients with *DOCK8* deficiency while pending transplant [79,80].

### 1.6. Mast Cell Degranulation

#### *PLCG2* GOF

**Figure 5:**
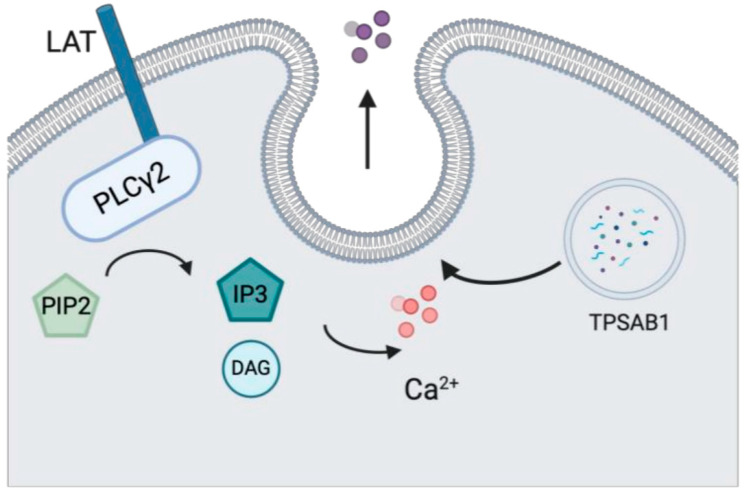
Mast cell degranulation may be triggered easily in PLCγ2 mutations, leading to spontaneous calcium influx that results in degranulation by sub-physiologic temperatures of all hematopoietic cells, except T cells.

*PLCG2* encodes phospholipase C gamma 2 (PLCγ2), a vital transmembrane signaling molecule particularly important in B lymphocytes, mast cells, and natural killer cells (Figure 5) [81]. Several complex dominant variants of *PLCG2* cause dysregulation at the autoinhibitory domain of PLCγ2, leading to spontaneous calcium flux and degranulation at sub-physiologic temperatures in nearly all hematopoietic cells, apart from T cells [81]. PLCγ2-associated antibody deficiency and immune dysregulation (PLAID) is an autosomal dominant primary immunodeficiency with onset in infancy [82]. It is characterized by cold urticaria with evaporative cooling, abnormal leukocyte signaling, hypogammaglobulinemia, recurrent sinopulmonary infections, autoimmunity, skin granulomas, and chronic inflammation [83]. Atopy, including allergic rhinitis, food allergies, asthma, and atopic dermatitis, is frequently observed in these patients [83]. The onset of symptoms varies but can occur as early as infancy [83]. The primary approach to treating these patients focuses on avoidance of evaporative or systemic cooling [82]. The addition of antihistamines can be effective as well in mitigating symptoms. For patients with immune dysregulation, antibiotic prophylaxis or immunoglobulin replacement therapy can be considered [82].

### 1.7. Cytokine Signaling

#### 1.7.1. *IL4RA* GOF

**Figure 6:**
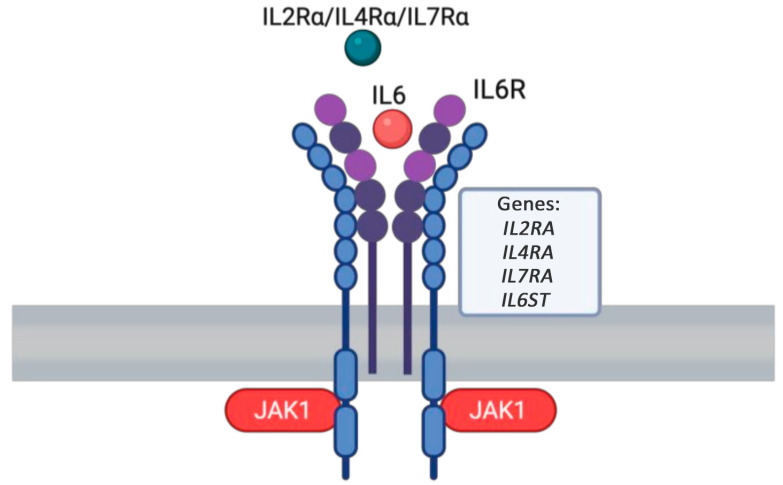
Cytokine signaling gets activated by various interleukins, which are critical for immune and inflammation regulation.

The R576 allele of the interleukin-4 receptor alpha (*IL4RA*) is strongly linked to atopy [84]. This variant alters the signaling function of the IL-4 receptor [84]. IL-4RA is a key subunit of the interleukin-4 receptor complex, critical for immune responses and inflammation regulation (Figure 6) [84]. In *IL4RA* GOF variants, there is an abnormal increase in receptor activity and responsiveness to IL-4 and potentially other cytokines that signal through this receptor [85]. This heightened signaling can disrupt immune responses, leading to chronic inflammation and symptoms resembling autoimmune diseases [84]. Clinically, *IL4RA* GOF variants are associated with atopy, hyper-IgE syndrome, and other immune dysregulation disorders [86,87]. These conditions often present with recurrent bacterial infections, elevated IgE levels, dermatitis, food allergies, and occasionally features resembling autoimmune conditions [87,88]. A cohort study conducted in school-aged children reported the mean age of patients to be around 8 years old; however, the onset of atopy was not reported [87]. Dupilumab has shown efficacy in reducing atopic symptoms in patients with hyper-IgE syndrome and other atopic disorders associated with *IL4RA* GOF variants [89].

#### 1.7.2. *IL2RA* LOF

The *IL2RA* gene encodes the interleukin-2 receptor alpha chain, which plays a crucial role in immune regulation and is implicated in various immune-mediated conditions, including those affecting the skin (Figure 6) [90]. Genetic variations or the dysregulation of *IL2RA* have been linked to atopic dermatitis [91]. Biallelic loss-of-function (LOF) variants in the alpha subunit of *IL2RA* (CD25), which forms a high-affinity IL-2 receptor with IL2RB and CD132, lead to an autosomal recessive immunodeficiency characterized by autoimmunity and eczema [92]. Autosomal recessive variants in *IL2RA* (CD25) are associated with a disease resembling IPEX syndrome, featuring autoimmune cytopenia and eczematous dermatitis [93]. Additionally, defects or variants in *IL2RA* may confer susceptibility to viral infections and different types of leishmaniasis, including visceral and cutaneous forms [94,95]. Suspicion of *IL2RA* deficiency arises in patients with recurrent infections including viral infections, autoimmunity, and lymphoproliferation [93,95]. A case report described a patient who developed a vaccine reaction at 1 year old [95]. Diagnosis involves immunologic testing, including flow cytometry to assess T, B, and NK cell populations, and detecting reduced or absent CD25 (*IL2RA*) expression on T cells, followed by genetic confirmation of the variant [94,96]. The primary curative treatment is HSCT, which should be performed as early as possible to prevent severe infections and complications [97].

#### 1.7.3. *IL6ST* LOF

Altered *IL6ST* signaling can exacerbate inflammatory skin conditions, such as atopic dermatitis [98]. Selective loss-of-function variants in *IL6ST* are associated with hyper-IgE syndrome, leading to distinct impairments in T cell phenotype and function and recessive forms of hyper-IgE syndrome with eosinophilia and atopic dermatitis [99,100]. A case report detailed a patient with *IL6ST* LOF who presented with recurrent bacterial infections, eczema, bronchiectasis, high IgE levels, and eosinophilia [101]. The patient developed atopic dermatitis at 2 years old [101]. Management includes infection prevention with prophylactic antibiotics and vaccinations and immune support through immunoglobulin replacement [88]. For severe cases, HSCT offers a potential cure by reconstituting the immune system [88].

#### 1.7.4. *IL6R* LOF

*IL6R* LOF variants can lead to atopic dermatitis, elevated IgE, bacterial sinopulmonary infections, and substantial skin and soft tissue infections [102]. Patients with *IL6R* variants may experience frequent infections and chronic skin inflammation due to impaired immune regulation [103]. Certain *IL6R* variants are associated with hyper-IgE syndrome [104]. The onset of atopy has been reported as early as 6 months of age [103]. Identifying specific *IL6R* variants in patients can lead to more personalized treatment approaches, improving outcomes for those with inflammatory skin diseases [105]. Immunoglobulin replacement therapy for patients with recurrent bacterial infections, hypogammaglobulinemia, and antimicrobial prophylaxis are the mainstays of treatment [106]. For severe cases with significant immunodeficiency and recurrent life-threatening infections, HSCT may be considered [107].

#### 1.7.5. *IL7RA* LOF

*IL7RA* encodes IL-7Rα, which is a part of the IL-7 receptor and is crucial for the homeostasis of lymphocytes, particularly T cells (Figure 6) [108]. Thymic stromal lymphopoietin (TSLP) is a cytokine that promotes the differentiation of type 2 helper T cells [109]. The IL-7Rα chain interacts with TSLP and the TSLP receptor and activates the intracellular signaling of the JAK/STAT pathway [110]. An SNP in TSLP is associated with the prevalence and persistence of atopic dermatitis [111,112]. Additionally, variants in the IL7R gene may modulate the effect of TSLP variants. For instance, the association of the TSLP SNP rs10073816 with atopic dermatitis persistence is strengthened when controlling for the IL7R SNP rs11567725 [112]. This relationship was explored in a large longitudinal cohort of individuals with atopic dermatitis to gain a more comprehensive understanding of their association with atopic dermatitis persistence [112]. The average age of atopy onset has been reported to be around 2 years [112]. IL7R variants have also been identified as risk factors for several diseases characterized by autoimmune or excessive immune and inflammatory responses, including multiple sclerosis, type 1 diabetes, and atopic dermatitis [90]. The primary treatment for IL7R deficiency is HSCT [113]. Management also includes supportive care, such as infection prevention with prophylactic antibiotics, antivirals, and antifungals; immunoglobulin replacement therapy; and close monitoring of immune function and protentional complications [113,114].

### 1.8. TGF-β Signaling

#### 1.8.1. *TGFBR1/2* LOF

Variants in both *TGFBR1* and *TGFBR2* cause Loeys–Dietz syndrome, a genetic disorder characterized by vascular abnormalities, skeletal deformities, and connective tissue issues [115,116]. *TGFBR1/2* variants can also lead to persistent, itchy, and inflamed skin, consistent with atopic dermatitis [117,118]. These variants in *TGFBR1/2* disrupt skin barrier homeostasis, increasing susceptibility to allergens and irritants, thereby worsening atopic dermatitis [118]. Additionally, *TGFBR1/2* variants are linked to other atopic conditions, such as asthma and allergic rhinitis, due to the crucial role of TGF-β signaling in immune regulation [119]. No studies have reported the onset age of atopy. However, one study examining the TGFB1 gene in patients with atopic dermatitis found that the mean age of these patients was 8 years, with the youngest being 1 year old [120]. Genetic testing confirms the variant, while imaging is important to monitor the extent of vascular and skeletal abnormalities [121]. Treatment primarily involves HSCT for immune restoration, along with supportive care to prevent infections and close monitoring of anatomic abnormalities [118]. Treatment can also include medical management with beta blockers and angiotensin receptor blockers to reduce cardiovascular stress and the risk of aortic dissection [122]. Surgical intervention is sometimes required for significant vascular or skeletal issues that impair function or quality of life [122,123].

#### 1.8.2. *ERBB2IP* LOF

*STAT3* negatively regulates TGF-β signaling via ERBIN (ERBB2-interacting protein) (Figure 2b) [124]. Cell-intrinsic deregulation of the TGF-β pathway promotes the IL-4/IL-4Rα/GATA3 axis, supporting atopic phenotypes in humans, including atopic dermatitis [124,125]. ERBB2 deficiency has clinical similarities to hyper-IgE syndrome [124]. There is no study reporting the onset of atopy. ERBIN anchors SMAD proteins, the main signal transducers for TGF-β receptors. This led to the discovery that *STAT3* negatively regulates TGF-β signaling through ERBIN [124]. A heterozygous LOF variant in *ERBB2IP*, encoding ERBIN, might be responsible for significant eosinophilic gastrointestinal diseases (EGIDs), allergen-specific reactivity, and connective tissue abnormalities [124]. A case report described the successful treatment for severe atopic dermatitis in this disease with dupilumab [126].

### 1.9. Skin Barrier

#### *SPINK5* LOF

**Figure 7:**
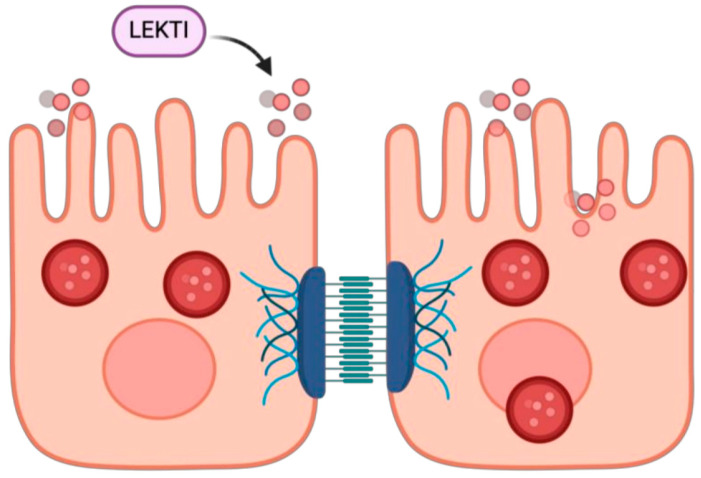
The skin barrier is maintained by several components, such as keratinocytes, intercellular adhesion proteins, and protease inhibitors, like *SPINK5*.

*SPINK5* (Serine Peptidase Inhibitor, Kazal Type 5) encodes the protein LEKTI (Lympho-Epithelial Kazal-Type Related Inhibitor), which is crucial for skin barrier function and immune response regulation (Figure 7) [127]. LEKTI inhibits various proteases that degrade structural proteins in the stratum corneum, the outermost layer of the skin [127]. By regulating protease activity, LEKTI maintains skin barrier integrity [127]. Deficiency in LEKTI due to *SPINK5* variants results in increased protease activity, leading to skin barrier disruption, increased transepidermal water loss, and enhanced penetration of allergens and irritants [127]. Variants in *SPINK5* are linked to Netherton syndrome, a rare genetic disorder characterized by severe skin disease, hair abnormalities, and atopic manifestations, including atopic dermatitis [128]. A study reported the onset of atopic dermatitis commonly occurs during infancy, at or before 2 years of age [129]. In a meta-analysis published in 2020, the *SPINK5* Asn368Ser polymorphism was identified as a potential risk factor for atopic dermatitis alone [130]. Patients with *SPINK5* variants often present with erythroderma (widespread redness and desquamation of the skin), ichthyosis linearis circumflexa (red, scaly plaques with double-edged scaling), and trichorrhexis invaginata (hair shaft abnormality known as “bamboo hair”) [131,132]. These patients commonly experience skin issues, such as refractory atopic dermatitis, and other allergic manifestations, including asthma, allergic rhinitis, and food allergies [133]. For immune defects, patients with Netherton syndrome have been shown to have humoral immune defects and impaired cellular responses (particularly NK cell function defect) with recurrent bacterial infections, particularly skin infections due to skin disruption [134]. The management of conditions related to *SPINK5* variants involves immunoglobulin replacement therapy, and intensive skin care management, which have shown significant improvement in patient outcomes [135]. Immunoglobulin replacement therapy has been demonstrated to improve patient outcomes [134,136]. Recent studies have also shown promising results with biologic treatments, including dupilumab and Ustekinumab [137,138,139,140].

### 1.10. Lymphocyte Development

#### 1.10.1. *RAG1/2* LOF

**Figure 8:**
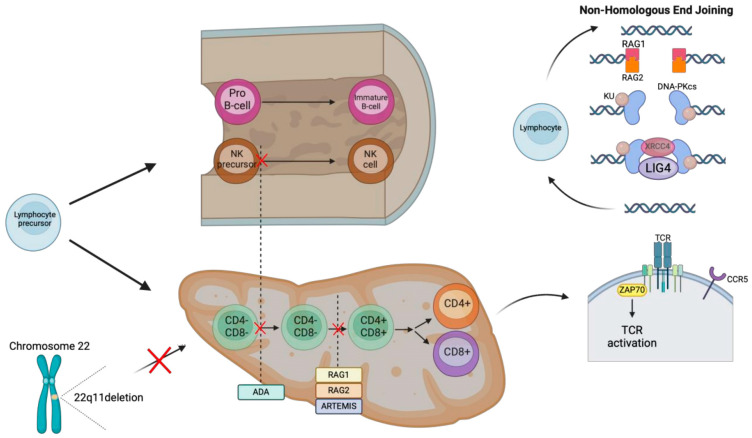
Lymphocyte development involves many significant proteins, such as *RAG1*&2, which are required for appropriate V(D)J recombination, in which mutations can lead to both B and T cell maturation failure.

The *RAG1* and *RAG2* proteins play a crucial role in V(D)J recombination, and in absent protein function, the patient develops severe combined immunodeficiency (SCID) with no mature functional T or B cells (Figure 8) [141,142]. However, if the protein is expressed hypomorphically, the same variant could lead to Omenn syndrome [143,144]. Common clinical presentations include recurrent sinopulmonary infections, atopic dermatitis, diarrhea, and oral thrush [145]. Atopic dermatitis can present as early as the first month of life [145]. High suspicion of IEIs is needed, as patients with the *RAG1* variant could have an atypical later-onset presentation, such as CVID, pyoderma gangrenosum, or even lymphoma [146,147,148,149]. Patients are diagnosed by genetic sequencing, and there are also functional studies confirming the absence of T and B cells on flow cytometry and oligoclonal T cell receptors [150]. T cell receptor excision circles (TRECs) by a polymerase chain assay on dried newborn blood spots through newborn screening can identify most cases of SCID when performed [151,152]. The standard treatment is HSCT [153].

#### 1.10.2. *DCLRE1C* LOF

Genetic variants in *DCLRE1C,* which encodes ARTEMIS, a nuclease necessary for opening the hairpin during V(D)J recombination, could lead to maturation failure of both B and T cells and the development of T-B-NK+ SCID similar to *RAG1/2*-deficient SCID (Figure 8) [154,155]. Common clinical manifestations of this disease include recurrent respiratory tract infections, diarrhea, and food allergies [156]. Food allergies can present as early as 2 months of age [156]. More recent studies have demonstrated that the phenotype variation of the *DCLRE1C* variant could also present as hyper-IgM syndrome, Omenn syndrome, chronic inflammatory bowel disease, or only antibody deficiency [157,158,159,160]. The mainstay of treatment for *DCLRE1C* variants is HSCT [161].

#### 1.10.3. *ADA* LOF

Adenosine deaminase (ADA) is the key enzyme in the purine metabolism pathway with its complete deficiency causing T-B-NK-SCID due to the accumulation of the toxic metabolites that block normal DNA synthesis and the development of lymphocytes (Figure 8) [151,162]. Partial ADA deficiency can cause later-onset immunodeficiency or no clinical manifestations at all [163,164]. Atopy and elevated IgE can be found in patients with ADA deficiency [165]. Patients can present with atopy as early as 6 months of age [165]. From the need for ADA throughout the body, ADA deficiency can also manifest with non-immune presentations, including hepatic dysfunction, sensorineural hearing loss, motor dysfunction, skeletal dysplasia, and cognitive and behavioral problems [166,167,168,169,170]. Tandem mass spectrometry measuring ADA levels can identify patients with late-onset ADA deficiency, which might not be identifiable with neonatal screening [171]. HSCT is the standard treatment for SCID from ADA deficiency [172,173]. Enzyme replacement therapy is also an alternative option [174]. Recently, retroviral and lentiviral gene therapies have shown promising results as an alternative option when HSCT is not available [175,176,177].

#### 1.10.4. *LIG4* LOF

DNA ligase IV (*LIG4*) deficiency is another example of T-B-NK+ SCID due to defective V(D)J recombination (Figure 8) [178]. *LIG4* is the main player in the final step of DNA repair using the non-homologous end joining (NHEJ) pathway, which is effective against double-strand breaks in which failure to repair could lead to aberrant V(D)J recombination [178,179]. The clinical presentation of *LIG4* deficiency can range from severe combined immunodeficiency to malignancy without overt immunodeficiency [180,181]. Patients can also have characteristic “bird-like” or “Seckel-like” facial dysmorphic, microcephaly, developmental retardation, and skin problems, including eczema [182]. Atopic dermatitis can develop as early as 1 month of age [183]. In addition to antimicrobial prophylaxis against opportunistic infections along with immunoglobulin replacement therapy, HSCT is a curative treatment of choice for patients with *LIG4* deficiency [184].

#### 1.10.5. *ZAP70* LOF

ZAP70 is one of the key regulatory proteins responsible for T cell receptor signaling (Figure 8), with its deficiency from the autosomal recessive genetic variants leading to a selective T cell deficiency with unique diagnostic characteristics of low CD8+ T cells and normal but non-functioning CD4+ T cells [185,186]. While a newborn screening with TREC is helpful for the diagnosis of most SCIDs, TREC levels in *ZAP70* LOF disease may not decrease below the screening threshold [187,188]. Patients with *ZAP70* deficiency can present with SCID, asthma, and atopic dermatitis, similar to Omenn syndrome [189]. Asthma and atopic dermatitis can present as early as 3 months of age [189]. Immunological evaluations typically reveal elevated IgE levels and eosinophilia [189]. Patients with the *ZAP70* variant respond well to HSCT with a promising survival rate [190].

#### 1.10.6. Deletion of Chromosome 22q11.2

Deletion of chromosome 22q11.2, or DiGeorge syndrome, is classically described in patients with poorly developed or absent thymus and parathyroid glands (Figure 8) [191]. There is a high variability of phenotypes, with the most common features including thymic and parathyroid hypoplasia or aplasia, with congenital heart defects leading to the unique characteristics of T cell immunodeficiency with hypocalcemia [192]. Patients with chromosome 22q11.2 deletion can also present with antibody deficiency or autoimmunity [193]. Atopy is common in patients with chromosome 22q11.2 deletion with a gradual skewing towards the Th2 phenotype [194]. Atopic dermatitis can present as early as 3 months of age [195]. While fluorescence in situ hybridization (FISH) is commonly used for the diagnosis of chromosome 22q11.2 deletion, some variations of deletion cannot be detected by this method [196]. Thus, diagnostic tools, such as the multiplex ligation-dependent probe amplification (MLPA) assay, can identify additional cases of chromosome 22q11.2 deletion [197]. Recently, cell-free DNA testing has shown promising results for pre-natal diagnosis [198]. Most patients with 22q11.2 deletion do not need immunoglobulin replacement therapy [199]. The importance of primary prevention with vaccines and antimicrobial prophylaxis is emphasized in the recent clinical practice guidelines [200]. Patients with congenital athymia have more significant life-threatening infections in addition to autoimmunity that can present with an oligoclonal T cell Omenn syndrome and dermatitis, and a thymic implant is the treatment of choice, which shows better survival rates than HSCT [201,202].

### 1.11. Regulatory T Cell

#### *FOXP3* LOF

**Figure 9:**
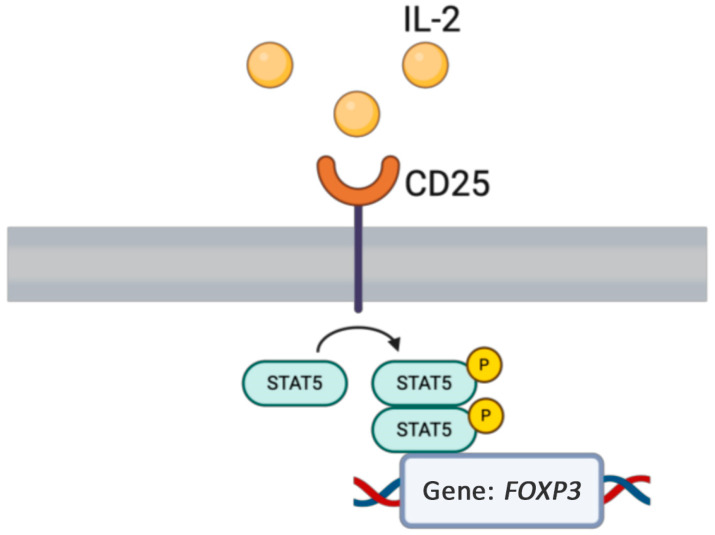
Regulatory T cell signaling requires the STAT5 pathway to encode the *FOXP3* gene, which is needed for appropriate T cell functioning and regulatory T cell differentiation.

Forkhead Box P3 (*FOXP3*) is crucial for sustaining the proper function and differentiation of regulatory T cells in which the variant of *FOXP3* leads to immune dysregulation, polyendocrinopathy, enteropathy, and X-linked (IPEX) syndrome (Figure 9) [203]. Patients typically present with diffuse atopic dermatitis, neonatal type 1 diabetes mellitus, and early-onset refractory diarrhea, along with variations of blood, kidney, liver, lung, and neuromuscular involvement [204,205]. One study reported the median age of disease onset to be 2 months [204]. The *FOXP3* variant can be diagnosed by a polymerase chain reaction (PCR) for the entire coding sequence of *FOXP3* or targeted Sanger sequencing [206]. The only curative treatment available for the *FOXP3* variant is HSCT, while immunosuppressive treatment, including myeloablative therapy, can provide temporary improvements [204].

### 1.12. Glycosylation

#### *PGM3* LOF

**Figure 10:**
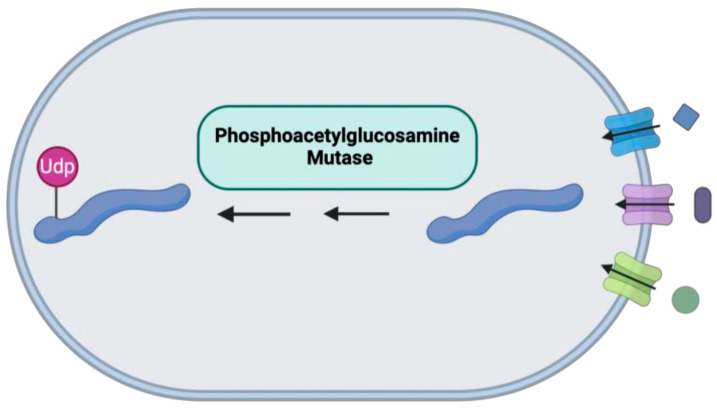
Mutations in the Phosphoacetylglucosamine Mutase 3 can lead to improper glycosylation, resulting in various conditions, such as hyper-IgE syndrome-like findings.

The significance of glycosylation in IEIs was emphasized when the case series reported the novel homozygous phosphoglucomutase 3 (*PGM3*) variants leading to hyper-IgE syndrome-like findings in children from consanguineous families (Figure 10) [207]. Patients can present with recurrent respiratory tract infections, skin infections, atopic dermatitis, asthma, and food allergies [208]. Atopic dermatitis and food allergies can occur as early as one year of age [208]. Moreover, a more recent case report of homozygous *PGM3* variants mentioned the development of bullous pemphigoid along with facial dysmorphism in addition to combined immunodeficiency [209]. In another report, two siblings with *PGM3* LOF disease demonstrated elevated IgE levels, atopic dermatitis, and CD4+ lymphopenia without dysmorphic features [210]. The diagnosis of *PGM3* deficiency can be made by genetic sequencing [211]. The main treatments are immunoglobulin replacement therapy and HSCT, along with antimicrobial prophylaxis [209,211,212].

## 2. Conclusions

The intersection between IEIs and severe early-onset atopy represents a complex and multifaceted area of clinical immunology. Allergic conditions are becoming more prevalent in the general population. As a result, IEIs can be overlooked if allergic symptoms, like atopic dermatitis, are the initial presentation, potentially leading to diagnostic delays. Patients with IEIs often present with atopic disorders that can be severe and resistant to standard therapies, highlighting the importance of recognizing the potential for underlying immunodeficiency in these cases. The genetic variants that underlie IEIs can disrupt immune regulation, leading to an imbalance in T-helper cell responses and promoting a Th2-skewed immune profile. These findings underscore the necessity for healthcare providers to consider IEIs in the differential diagnosis when faced with patients presenting with atypical or severe allergic manifestations.

Future studies should emphasize the diverse clinical presentations of atopy in the context of IEIs, as these can vary widely among individuals, ranging from mild allergic symptoms to severe and life-threatening reactions. Recognizing the spectrum of atopic manifestations is crucial for clinicians, as early diagnosis of IEIs can significantly alter the course of treatment and improve patient outcomes. Immunological testing should be considered for patients presenting with severe, atypical, or treatment-resistant atopic symptoms, recurrent infections, or a family history of IEIs. Prompt referral to an allergist and immunologist when IEIs are suspected is vital for obtaining an accurate diagnosis and initiating appropriate management strategies. Such referrals enable timely interventions, such as infection prevention, immunoglobulin replacement therapy, and hematopoietic stem cell transplantation (HSCT), which can offer curative potential for certain IEIs. HSCT has been shown to restore immune function and reduce atopic symptoms, underscoring its importance as a treatment option for severe cases. A comprehensive understanding of the relationship between IEIs and atopy, combined with advances in genetic screening and diagnostic tools, will enable healthcare providers to tailor management strategies more effectively, ultimately enhancing the quality of life for patients with these complex conditions.

## Figures and Tables

**Table 1:** Summary of inborn errors of immunity presenting with severe early-onset atopy.

Genes	Inheritance	Clinical Presentations	Managements
T cell signaling			
*CARD11* GOF	AD	CID, asthma	Immunoglobulin replacement, treatment of infections
*CARD11* LOF	AD/AR	CID, severe atopic dermatitis, atopic dermatitis, urticaria, recurrent infections	Immunoglobulin replacement, treatment of infections
*CARD11* DN	AD	CID, severe atopic dermatitis, viral skin infections, lymphoma	Immunoglobulin replacement, treatment of infections
*CARD14* GOF	AD	Inflammatory skin features (particularly psoriasis)	Ustekinumab
*CARD14* LOF	AD	Severe atopic dermatitis	Ustekinumab
*MALT1* LOF	AR	Atopic dermatitis, recurrent infections, failure to thrive	Immunoglobulin replacement, HSCT
*CARMIL2* LOF	AR	CID, atopic dermatitis, esophagitis, recurrent skin and chest infections	Immunoglobulin replacement, HSCT
JAK/STAT pathway			
*STAT1* GOF	AD	Atopic dermatitis, recurrent mucocutaneous fungal infections, recurrent viral infections	Long-term antifungal therapy, JAK inhibitors, HSCT
*STAT3* DN	AD	Hyper-IgE syndrome, atopic dermatitis, recurrent skin infections	Infection prophylaxis, HSCT
*STAT5b* LOF	AR	Atopic dermatitis, growth hormone insensitivity	Immunosuppressive therapy, hormone therapy
*STAT5b* GOF	AD	Severe atopic dermatitis, chronic urticaria, alopecia, angioedema, gastrointestinal symptoms	JAK inhibitors, HSCT
*JAK1* GOF	AD	Severe atopic dermatitis, asthma, food allergies, failure to thrive, autoimmune thyroiditis, hypothyroidism, gastrointestinal symptoms	JAK inhibitors
*ZNF341* LOF	AR	Atopic dermatitis, recurrent skin infections, chronic mucocutaneous candidiasis	Supportive treatments for infections and atopic dermatitis
NFKB			
*RelB* LOF	AR	Recurrent bacterial infections, atopic dermatitis, asthma, failure to thrive	Immunoglobulin replacement, infection prophylaxis, HSCT
*NF-κB1* LOF	AR	CID, atopic dermatitis, recurrent respiratory infections, recurrent skin infections, EBV proliferations	Immunoglobulin replacement, steroids, HSCT
*IKBKG* LOF	XL/AD	Hypodontia, hypohidrosis, hypotrichosis, atopic dermatitis-like rash, abnormal facial features	HSCT
Cytoskeletal pathway			
*WAS* LOF	XL	CID, thrombocytopenia, atopic dermatitis, recurrent infections, lymphoma	HSCT
*WIPF1* LOF	AR	CID, thrombocytopenia, atopic dermatitis, recurrent infections, lymphoma	HSCT
*ARPC1B* LOF	AR	CID, atopic dermatitis, food allergies, asthma	HSCT
*DOCK8* LOF	AD	CID, atopic dermatitis, asthma, severe food/environmental allergies, severe viral skin infection, bacterial respiratory infection, malignancies	Immunoglobulin replacement, HSCT, dupilumab
Mast cell degranulation			
*PLCG2* GOF	AD	CID, cold urticaria, recurrent sinopulmonary infections, skin granulomas, allergic rhinitis, food allergies, asthma, atopic dermatitis	Avoid evaporative/systemic cooling, antihistamines, immunoglobulin replacement, infection prophylaxis
Cytokine signaling			
*IL4RA* GOF	AD	Atopic dermatitis, food allergies, recurrent infections	Dupilumab
*IL2RA* LOF	AR	Atopic dermatitis, recurrent infections (viral infections, leishmanisis), autoimmune cytopenias,	HSCT
*IL6ST* LOF	AR	Atopic dermatitis, recurrent bacterial infections, bronchiectasis	Immunoglobulin replacement, infection prophylaxis, HSCT
*IL6R* LOF	AR	Atopic dermatitis, recurrent bacterial infections	Immunoglobulin replacement, infection prophylaxis, HSCT
*IL7RA* LOF	AR	Atopic dermatitis, autoimmune diseases (multiple sclerosis, type 1 diabetes)	Immunoglobulin replacement, infection prophylaxis
TGF-β signaling			
*TGFBR1/2* LOF	AD	Atopic dermatitis, asthma, allergic rhinitis, vascular abnormalities, skeletal deformities, connective tissue diseases, intestinal inflammation	HSCT, beta blockers, ARBs, surgeries for vascular and skeletal deformities
*ERBB2IP* LOF	AD	Atopic dermatitis, asthma, recurrent sinopulmonary infections connective tissue diseases, eosinophilic gastrointestinal diseases	Dupilumab
Skin barrier			
*SPINK5*	AR	Erythroderma, ichthyosis, bamboo hair (hair shaft abnormality), atopic dermatitis, asthma, allergic rhinitis, food allergy, recurrent bacterial infections	Skin hydration, topical steroids, topical calcineurin inhibitors, immunoglobulin replacement, biologics
Lymphocyte development			
*RAG1/2* LOF	AR	Omenn syndrome, recurrent sinopulmonary infections, atopic dermatitis, diarrhea, oral thrush, SCID, pyoderma gangrenosum, lymphoma	Immunoglobulin replacement, HSCT
*DCLRE1C* LOF	AR	Omenn syndrome, hyper-IgM syndrome, SCID, recurrent sinopulmonary infections, food allergies chronic inflammatory bowel disease, antibody deficiency	Immunoglobulin replacement, HSCT
*ADA* LOF	AR	SCID, atopic dermatitis, food allergy, asthma, hepatic dysfunction, neurosensory hearing loss, skeletal dysplasia, cognitive problems	Enzyme replacement therapy, HSCT
*LIG4* LOF	AR	SCID, atopic dermatitis, bird-like or Seckel-like facial dysmorphic, microcephaly, developmental delay, malignancy	Immunoglobulin replacement, infection prophylaxis, HSCT
*ZAP70* LOF	AR	SCID, asthma, atopic dermatitis	HSCT
22q11.2 deletion syndrome	AD	DiGeorge syndrome, SCID, atopic dermatitis, thymic hypoplasia, parathyroid hypoplasia, congenital heart defects	Infection prophylaxis, vaccinations, thymus transplantation, HSCT
Regulatory T cell			
*FOXP3* LOF	XL	IPEX syndrome, atopic dermatitis, polyendocrinopathy, type 1 diabetes mellitus, refractory diarrhea	Immunosuppressive therapy, HSCT
Glycosylation			
*PGM3* LOF	AR	Hyper-IgE syndrome, CID, atopic dermatitis, asthma, food allergies, recurrent respiratory/skin infections, skeletal dysplasia, bullous pemphigoid	Immunoglobulin replacement, infection prophylaxis, HSCT

AD, autosomal dominant; AR, autosomal recessive; ARB, angiotensin receptor blocker; CID, combined immunodeficiency; DN, dominant negative; GOF, gain of function; HSCT, hematopoietic stem cell transplantation; IPEX syndrome, immune dysregulation, polyendocrinopathy, enteropathy X-linked syndrome; JAK, Janus kinase; LOF, loss of function; SCID, severe combined immunodeficiency; XL, X-linked.
